# Endoscopic findings of gastric mixed adenoneuroendocrine carcinoma: A case report

**DOI:** 10.1097/MD.0000000000022306

**Published:** 2020-09-18

**Authors:** Keitaro Takahashi, Mikihiro Fujiya, Takahiro Sasaki, Yuya Sugiyama, Yuki Murakami, Takuya Iwama, Takehito Kunogi, Katsuyoshi Ando, Nobuhiro Ueno, Shin Kashima, Kentaro Moriichi, Hiroki Tanabe, Sayaka Yuzawa, Hidehiro Takei, Toshikatsu Okumura

**Affiliations:** a Division of Gastroenterology and Hematology/Oncology, Department of Medicine, Asahikawa Medical University; b Department of Diagnostic Pathology, Asahikawa Medical University, Asahikawa, Japan.

**Keywords:** endoscopic submucosal dissection, magnifying endoscopy, MANEC, mixed adenoneuroendocrine carcinoma, narrow band imaging

## Abstract

**Rationale:**

Gastric mixed adenoneuroendocrine carcinoma (gMANEC) is a rare malignant tumor. Most gMANECs are diagnosed at an advanced stage and have a worse prognosis than gastric adenocarcinoma. In order to improve the prognosis, it is necessary to diagnose gMANEC at an early stage. However, the endoscopic features of early gMANECs are unclear. We, herein, report a case of early gMANEC that showed characteristic magnifying endoscopic findings.

**Patient concerns:**

A 78-year-old man was referred to our institution for endoscopic resection of a gastric lesion. He had a medical history of distal gastrectomy due to early gastric cancer with negative surgical margins 9 years previously.

**Diagnosis:**

Esophagogastroduodenoscopy showed a reddish depressed lesion on the suture line of the gastric remnant, which was classified as type 0-IIc according to the Paris classification. ME-NBI at the oral side of the lesion revealed the absence of the microsurface pattern (MSP) and scattered microvessels with dilation and caliber variation, while ME-NBI at the anal side showed an irregularly tubular MSP. An endoscopic forceps biopsy showed a well- to moderately differentiated adenocarcinoma.

**Interventions:**

We performed endoscopic submucosal dissection, and *en bloc* resection of the tumor was successfully achieved.

**Outcomes:**

The histological findings showed two distinct components: neuroendocrine carcinoma (NEC) and well-differentiated adenocarcinoma, which comprised ∼60% and 40% of the tumor, respectively. The NEC component corresponded to the site with the absence of an MSP and scattered microvessels on ME-NBI, while the well-differentiated adenocarcinoma component corresponded to the site with an irregularly tubular MSP. The pathological diagnosis was mixed adenoneuroendocrine carcinoma, infiltrating into the deep submucosal layer.

**Lessons:**

We propose that the absence of an MSP plus an irregular MSP is characteristics of gMANEC, which was useful for the diagnosis of gMANEC before treatment.

## 1 Introduction

According to the World Health Organization (WHO) 2010 classification, mixed adenoneuroendocrine carcinoma (MANEC) is defined as a tumor composed of adenocarcinoma and neuroendocrine carcinoma, each of which composes at least 30% of the lesion.^[1,2]^ In the WHO 2019 classification, MANECs and mixed adenocarcinoma-neuroendocrine tumors were included in an umbrella category of mixed neuroendocrine-non-neuroendocrine neoplasms (MiNENs).^[3]^ The epidemiology of gastric MANEC (gMANEC) has not yet been described due to its rarity, while gMANECs account for about 20% of all digestive MANECs.^[3]^ Based on a small series of patients and case reports, most gMANECs are diagnosed at an advanced stage^[1,4]^ and have a worse prognosis than gastric adenocarcinoma, with survival time usually measured in months.^[2–4]^ In order to improve the prognosis, the diagnosis of gMANEC at an early stage is needed; however, early-stage gMANECs in which invasion is limited to the submucosa are less frequently identified because of the low diagnostic ability of endoscopic biopsy.^[5,6]^ Additionally, the endoscopic features of early gMANEC, particularly the magnifying endoscopy with narrow band imaging (ME-NBI) features, are unclear. We herein report a case of early gMANEC that showed characteristic magnifying endoscopic findings.

## 2 Case report

**Figure 1:**
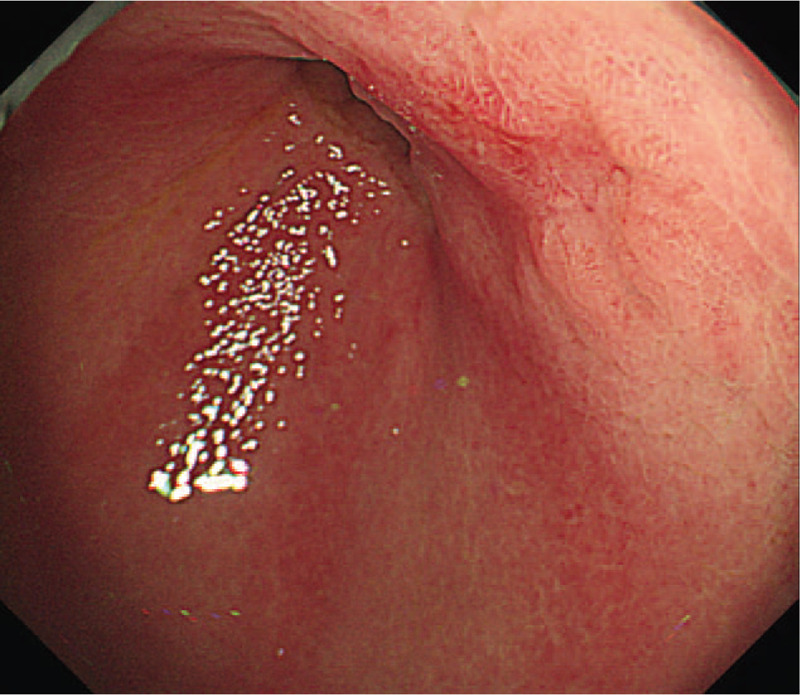
Conventional endoscopy findings. EGD showed a reddish depressed lesion on the suture line of the gastric remnant, classified as type 0-IIc according to the Paris classification.

**Figure 2:**
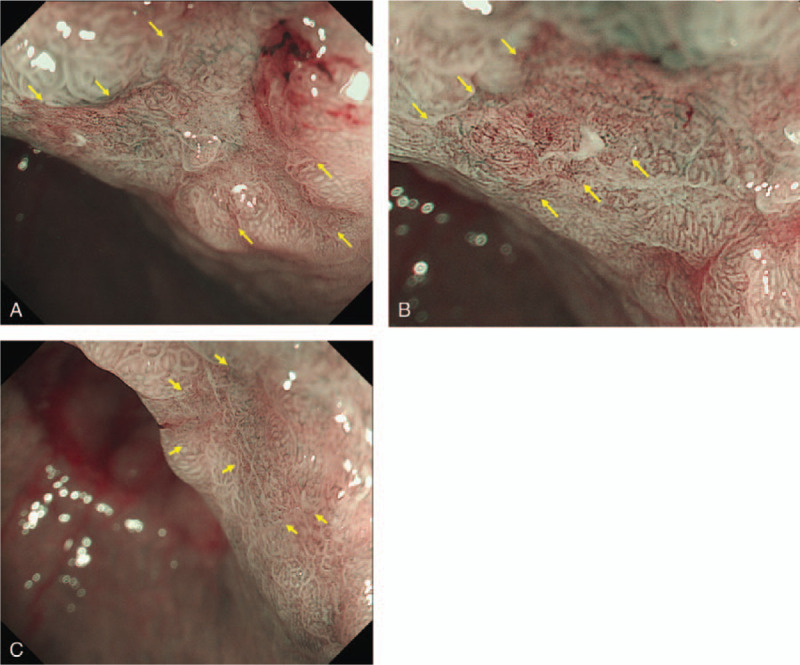
Magnifying endoscopy findings with narrow band imaging (ME-NBI). A demarcation line was noted around the 0-IIc lesion (A). ME-NBI on the oral side of the lesion revealed the absence of a microsurface pattern (MSP) and scattered microvessels with dilation and caliber variation (B). ME-NBI on the anal side an showed irregularly tubular MSP (C).

A 78-year-old man, who had undergone distal gastrectomy due to early gastric cancer with negative surgical margins nine years previously, underwent follow-up esophagogastroduodenoscopy (EGD), which was performed by his primary physician. EGD revealed a gastric tumor in the gastric remnant and endoscopic forceps biopsy showed a well- to moderately-differentiated adenocarcinoma. The patient was referred to our institution for endoscopic resection of the gastric lesion. A physical examination revealed no abnormal findings. The patient's blood test parameters were within the normal limits, including his carcinoembryonic antigen and cytokeratin 19 fragment levels. The patient was negative for serum IgG antibody and stool antigen to *Helicobacter pylori* (*H.pylori*), as *H.pylori* eradication had been achieved after the previous surgery. Computed tomography (CT) showed no evidence of lymph node metastasis. EGD showed a reddish depressed lesion on the suture line of the gastric remnant, which was classified as type 0-IIc according to the Paris classification (Fig. 1). On ME-NBI, a demarcation line (DL) was noted around the 0-IIc lesion (Fig. 2A). On the oral side of the lesion, ME-NBI revealed the absence of a microsurface pattern (MSP) and scattered microvessels with dilation and caliber variation (Fig. 2B). In contrast, on the anal side, ME-NBI showed an irregularly tubular MSP (Fig. 2C). Due to the absence of the MSP and the presence of an irregular MSP as well as the irregular microvascular pattern with a DL, we diagnosed the lesion as a well- to moderately-differentiated adenocarcinoma, while the histological findings of the area with absent MSP with scattered microvessels could not be predicted. Based on these endoscopic findings, we believed that the tumor was intramucosal gastric adenocarcinoma, for which removal by endoscopic submucosal dissection (ESD) was suitable. We performed ESD using a FlushKnife B25S device (FUJIFILM Medical Co., Ltd., Japan). Thereafter, the *en bloc* resection of the tumor was successfully performed.

**Figure 3:**
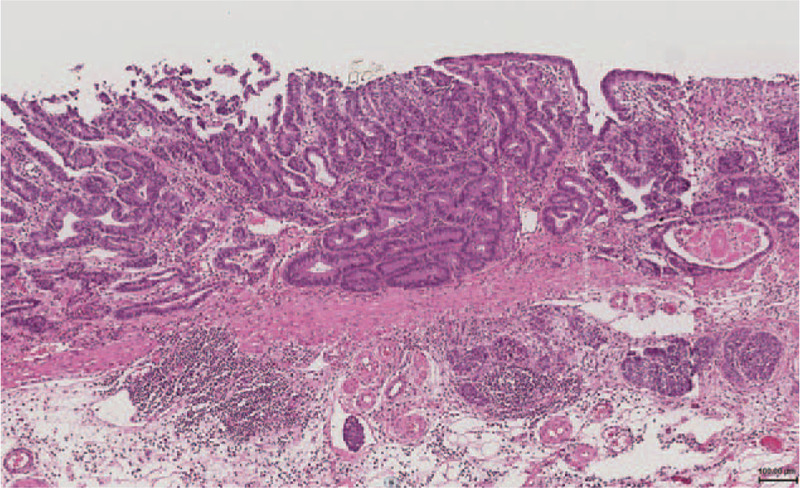
Histological findings. The invasive depth of the adenocarcinoma component was limited to the mucosal layer.

**Figure 4:**
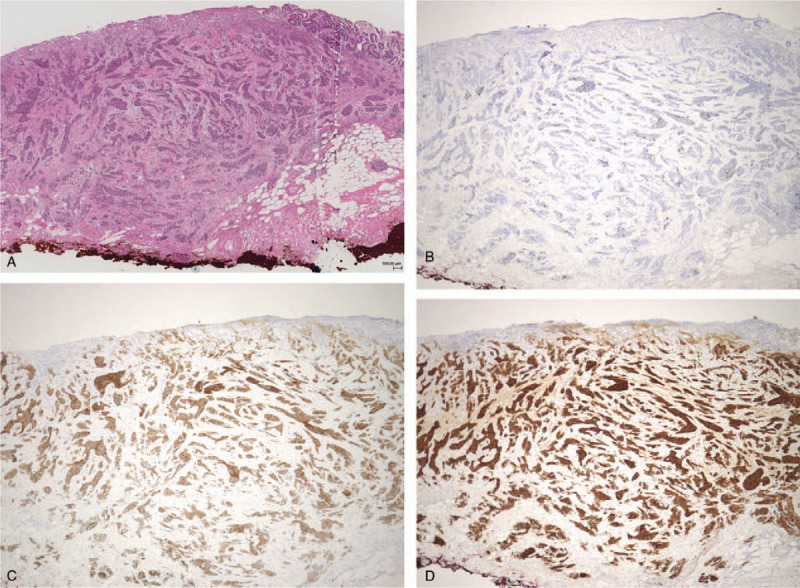
Immunohistochemistry findings. Hematoxylin-eosin stained sections of the NEC showed fusiform nuclei with granular chromatin and a nesting growth pattern, infiltrating into the deep submucosal layer (A). The NEC component was positive for chromogranin A (B), synaptophysin (C) and CD56 (D) from the surface of the mucosal layer to the deep submucosal layer.

The histological findings revealed that the tumor was composed of two distinct components, a neuroendocrine carcinoma (NEC) component and a well-differentiated adenocarcinoma component, which composed approximately 60% and 40% of the tumor, respectively. The NEC component corresponded to the site with the absence of an MSP and scattered microvessels on ME-NBI (Fig. 2B), while the well-differentiated adenocarcinoma component corresponded to the site of the irregularly tubular MSP (Fig. 2C). The invasive depth of adenocarcinoma was limited to the mucosal layer (Fig. 3). Lymphatic vessel invasion was seen on D2–40 and the vertical margin was positive for NEC. Hematoxylin-eosin stained sections of the NEC component revealed fusiform nuclei with granular chromatin and a nesting growth pattern, infiltrating into the deep submucosal layer (Fig. 4A). The mitotic activity was 3/10 high-power field (HPF) and the Ki-67 index of the NEC component was 50%. An immunohistological examination showed that the NEC component was positive for chromogranin A, synaptophysin and CD56 from the surface of mucosal layer to the deep submucosal layer (Fig. 4B–D). Based on these findings, the pathological diagnosis was mixed adenoneuroendocrine carcinoma, type 0-IIc, 14 × 11 mm, T1b2(SM2), UL1, ly1, v0, pHM0, pVM1. Total gastrectomy with lymph node dissection was performed; however, no residual cancer cells were observed. EGD and CT at 1 year after surgery revealed no local recurrence or lymph node metastasis.

## 3 Discussion

We reported a rare case of early gMANEC with characteristic findings of the absence of an MSP plus an irregular MSP on ME-NBI, which corresponded to the two components of NEC and adenocarcinoma. It is noteworthy that the area where the NEC component was exposed to the superficial epithelium was the site in which the MSP was absent and in which scattered microvessels were observed on ME-NBI (Fig. 2B and 4A–D). These are characteristic findings of the NEC component on ME-NBI. On the other hand, the area with an irregular MSP on ME-NBI histologically corresponded to the location of the well-differentiated adenocarcinoma component (Fig. 2C and 3). ME-NBI can therefore distinguish the NEC component from the adenocarcinoma component based on the characteristic findings of NEC, specifically, the absence of an MSP and scattered microvessels. This is the first report to identify the characteristic findings of NEC and adenocarcinoma components on ME-NBI, which are useful for the diagnosis of gMANEC.

Including the present case, only ten cases of gMANEC invasion limited to the submucosa have been reported^[1–2,5,7–12]^ (Table 1). These included 8 male and 2 female patients. All lesions were detected in the lower or middle third of the stomach. The median size of the lesions was 15.2 mm. According to the Paris classification, 9 lesions were morphologically classified as non-polypoid type without mixed type (0-IIa or 0-IIc) and 1 lesion was classified as polypoid type (0-I). Three of the 5 cases in which ME-NBI was performed showed the absence of an MSP plus an irregular MSP. Ours was the only case in which scattered microvessels were detected in the area near the NEC component. It is also recommended that forceps biopsy is performed to obtain a specimen from the part of the tumor with the absence of an MSP and scattered microvessels on ME-NBI.

**Table 1:**
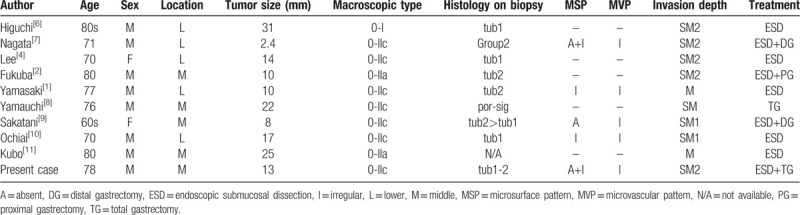
Cases of gastric mixed adenoneuroendocrine carcinoma.

The treatment of early gMANEC has not been established due to its rarity; however, most are treated with ESD according to the strategy for early gastric cancer.^[1,2,5,7,8,11–12]^ Eight lesions showed submucosal invasion, while two were intramucosal. Nine lesions were initially treated with ESD, with additional gastrectomy performed for four of these nine cases. Among the five patients who received ESD alone, two cases with submusocal invasion developed lymph node metastasis, liver metastasis, or peritoneal dissemination after ESD,^[11,13]^ suggesting the high risk of liver and/or lymph node metastasis in gMANEC, which is not an indication for ESD. Thus, the pre-operative diagnosis of gMANEC is important for appropriate treatment selection. ME-NBI might enable the diagnosis of gMANEC based on the characteristic findings. ME-NBI is also useful for identifying the appropriate site to perform endoscopic forceps biopsy.

In conclusion, we proposed—for the first time—that the absence of an MSP plus an irregular MSP on ME-NBI are characteristics of gMANEC. These findings may be useful for diagnosing gMANEC before treatment.

## Acknowledgments

No other individuals contributed to this manuscript.

## Author contributions

KT and MF conducted the study and wrote the initial draft of the manuscript. TS performed ESD. SY and HT provided a pathological diagnosis. YS, YM, and NU managed the patient. TI, TK, KA, and SK contributed to the analysis and interpretation of the manuscript. KM, HT, and TO have contributed to data collection and interpretation, and critically reviewed the manuscript. All authors approved the final version of the manuscript and agree to be accountable for all aspects of the work in ensuring that questions related to the accuracy or integrity of any part of the work are appropriately investigated and resolved.

## Abbreviations

Abbreviations: CT = computed tomography, DL = demarcation line, EGD = esophagogastroduodenoscopy, ESD = endoscopic submucosal dissection, gMANEC = gastric mixed adenoneuroendocrine carcinoma, *H. pylori* = *Helicobacter pylori*, MANEC = mixed adenoneuroendocrine carcinoma, ME-NBI = magnifying endoscopy with narrow band imaging, MSP = microsurface pattern, NEC = neuroendocrine carcinoma.
